# βIV-Spectrin in Cardiac Fibroblasts: Implications for Fibrosis and Therapeutic Targeting in Cardiac Diseases. Comment on Nassal et al. Spectrin-Based Regulation of Cardiac Fibroblast Cell-Cell Communication. *Cells* 2023, *12*, 748

**DOI:** 10.3390/cells12172186

**Published:** 2023-08-31

**Authors:** Wenjing Xiang, Ning Zhou, Lei Li, Faming Chen, Lei Li, Ying Wang

**Affiliations:** 1 Department of Pharmacology, School of Medicine, Southern University of Science and Technology, Shenzhen 518055, Chinachenfaming66@163.com (F.C.); 2 School of Public Health, Xi’an Jiaotong University, Xi’an 710061, China; 1007006846@stu.xjtu.edu.cn

Fibroblasts in the heart, traditionally recognized as interstitial cells, have long been overlooked in the study of cardiac physiology and pathology. However, recent advances have shed light on their essential role in sensing environmental stress stimuli and communicating with various cell types [1]. In pathological conditions, such as myocardial damage or inflammation, fibroblasts undergo increased proliferation and extracellular matrix secretion, thereby triggering cardiac fibrosis and remodeling. Targeting cardiac fibroblasts, rather than cardiomyocytes, offers an alternative therapeutic avenue for managing heart diseases.

βIV-spectrin, a prominently enriched membrane-associated cytoskeletal protein, serves as a key anchor and stabilizer of the signaling complex [2]. Prior investigations of βIV-spectrin within the heart predominantly centered on its physiological and pathological roles in cardiomyocytes, leaving much to be uncovered about its regulation and impact on cardiac fibroblasts—the predominant non-cardiomyocyte cell type in the heart [3]. Recent studies conducted by the same research group have added new knowledge, revealing that the absence of βIV-spectrin in fibroblasts triggers fibroblast activation and facilitates fibrotic gene transcription, consequently resulting in escalated fibrosis and impaired cardiac function [4]. Over past decades, it has been demonstrated that cell communication, heavily reliant on the dynamic interaction between the cell membrane and cytoskeleton, significantly shapes fibroblast function [5]. Yet, whether and how βIV-spectrin modulates fibroblast communication have remained unknown, representing a potential target for cardiac antifibrotic therapy. In the present study, Nassal et al. published the article entitled “Spectrin-Based Regulation of Cardiac Fibroblast Cell-Cell Communication” in *Cells* and provided answers to these crucial questions, uncovering that βIV-spectrin modulates fibroblast–fibroblast communication by facilitating the release of pro-fibrotic and pro-inflammatory exosomes within the heart [6].

In their research, the authors collected conditioned culture media (CCM) from WT and βIV-spectrin-deficient (qv^4J^) primary cardiac fibroblasts. Notably, media from (qv^4J^) fibroblasts were found to enhance fibroblast proliferation and collagen deposition, underscoring the essential role of cell–cell communication involving βIV-spectrin. To elucidate the pathogenic gradient in qv^4J^ fibroblast CCM, proteome profiler analysis was deployed, identifying an array of increased pro-inflammatory cytokines/chemokines and pro-fibrotic growth factors. These factors are known to enhance fibroblast activation and proliferation. Cell-derived exosomes critically mediate such paracrine-based cell communication and might account for the elevated secretion of pathologic cytokine/chemokines and growth factors. Indeed, exosomes are capable of transferring proteins, including cytokines and chemokines, to recipient cells and thus lead to phenotypic changes in the latter cell. Human cardiac fibroblast can release exosomes into the cell culture supernatant, and this phenomenon has been linked to cardiac diseases, including heart failure (HF). Therefore, extracellular vehicles in CCM were isolated and characterized using the NS300 nanoparticle tracking system. The results showed an increased concentration of spectrin-deficient cardiac-fibroblast-derived exosomes, aligning with the heightened fibroblast–fibroblast communication resulting from βIV-spectrin deficiency. It has been established that βIV-spectrin forms a complex with transcriptional factor STAT3, and the degradation of βIV-spectrin alters the nuclear localization of STAT3 in both cardiomyocytes and CFs. In this study, inhibiting STAT3 reduced concentrations of exosomes and pro-inflammatory and profibrotic cytokines/chemokines released by βIV-spectrin-deficient CFs. This research defines a novel role for βIV-spectrin/STAT3 in modulating fibroblast exosome release and the emission of fibrotic and inflammatory paracrine signals, thereby reshaping fibroblast–fibroblast communication. It introduces βIV-spectrin/STAT3 as a fresh target for cell–cell communication, further expanding our comprehension of the βIV-spectrin signaling pathway’s contributions to cardiac physiology and pathology.

While the recent findings by Nassal et al. are both captivating and broadening in terms of the role of βIV-spectrin signaling in modulating fibroblast–fibroblast communication, they also spark several thought-provoking scientific inquiries. These encompass the following: firstly, investigating the cell-type specificity of βIV-spectrin signaling within the heart; secondly, examining the role of βIV-spectrin signaling in less explored cardiac conditions such as heart failure with preserved ejection fraction (HFpEF); and lastly, exploring the development of therapeutic strategies targeting βIV-spectrin signaling.

Considering the cell-type specificity and similarities of βIV-spectrin is imperative to advance our understanding of βIV-spectrin signaling in the heart, given the multifunctional role of βIV-spectrin in distinct cell types. The deficiency of βIV-spectrin in either cardiomyocytes or fibroblasts can lead to cardiac dysfunction through distinct molecular mechanisms [3,4]. In cardiomyocytes, βIV-spectrin is essential for excitation–contraction coupling, while in fibroblasts, βIV-spectrin-mediated electrical signaling is unknown. The Na^+^ and K^+^ channels, which are functionally expressed in fibroblasts [7], can be affected through βIV-spectrin deletion. In pathological conditions, cardiac fibroblast hyperpolarization may involve βIV-spectrin dysregulation. Actually, fibroblast activation is regulated by Na^+^ current, which may couple with βIV-spectrin [2]. Therefore, it is plausible that cardiac-fibroblast-specific deletion of βIV-spectrin could lead to the ion channels’ dysfunction and contribute to cardiac fibrosis. In addition to fibroblasts and cardiomyocytes, βIV-spectrin may also critically affect other cell types, including endothelial cells and immune cells. Indeed, the endothelial-cell-specific loss of βIV-spectrin in mice results in hypervascularization and embryonic lethality. The impact of βIV-spectrin on cytokines/chemokines secretion indicates that βIV-spectrin may be a crucial regulator for immune cells such as macrophages. To uncover the cell-type-specific functions and signaling regulation of βIV-spectrin within the heart, a single-cell multiomics approach and cell-type-specific knockout animals, such as fibroblast-specific βIV-spectrin knockout mice, can be integrated in future studies.

CaMKII’s pivotal role in regulating βIV-spectrin has been established, although its involvement in fibroblast communication remains unexplored. The same research group elucidated a signalosome framework facilitated by βIV-spectrin, which encompasses CaMKII and the transcriptional factor STAT3. CaMKII-dependent phosphorylation of βIV-spectrin leads to its degradation, triggering a disruption in STAT3-mediated gene transcription [8]. In a hypertrophy-induced heart failure (HF) model, disrupting the βIV-spectrin-CaMKII interaction prevents CaMKII-induced degradation of spectrin, safeguarding the heart against hypertrophy-induced cardiac fibrotic remodeling and dysfunction. Impaired STAT3 activity underlies the profibrotic gene transcription, cardiac fibrosis, and reduced cardiac function observed in mice with cardiac-specific βIV-spectrin knockout (βIV-cKO). The present study unveils a fresh role for βIV-spectrin-STAT3 in mediating exosomes and fibroblast communication. In pathological conditions such as HF, CaMKII activation can be instigated through profibrotic TGFβ signaling, potentially engaging in fibroblast–fibroblast communication through phosphorylation of βIV-spectrin (as depicted in Figure 1).

It has been shown that βIV-spectrin dysregulation is involved in human HF, which is now classified into two subgroups, heart failure with reduced ejection fraction (HFrEF) and heart failure with preserved ejection fraction (HFpEF). Distinct to HFrEF, HFpEF accounts for more than 50% of HF patients, with its pathogenesis much less understood [9]. HFpEF is characterized by an excessive extracellular matrix (ECM) deposition and cardiac fibrosis, yet a viable therapeutic approach targeting HFpEF is yet to be established. Interestingly, upon a re-evaluation of transcriptome data from myocardial samples of HFpEF patients [9], a specific upregulation of βIV-spectrin mRNA levels was observed in HFpEF hearts (as depicted in Figure 2). This raises intriguing questions: Does this alteration of βIV-spectrin occur within the fibroblasts of HFpEF-affected hearts? And how might this βIV-spectrin dysregulation contribute to fibrosis in HFpEF? Addressing these inquiries holds the potential to advance our comprehension of βIV-spectrin’s role in cardiac fibroblast biology, warranting further dedicated investigation.

Targeting βIV-spectrin for potential cardiac gene therapy holds promise as a therapeutic strategy. The observed induction of cardiac fibrosis and remodeling upon deletion of βIV-spectrin aligns with the decreased levels of βIV-spectrin in failing hearts. Consequently, βIV-spectrin emerges as a viable therapeutic target for heart failure. The advancement of cardiac gene editing techniques, such as gene-encoded adeno-associated virus delivery, CRISPR-Cas9-mediated gene deletion, overexpression, and modified RNA-based gene therapy, offers various effective approaches for manipulating βIV-spectrin in the heart [10,11]. Determining the most appropriate and efficient gene therapy approach to alleviate cardiac fibrosis and restore cardiac function in humans is of significant interest. Further investigations are necessary to evaluate and compare the efficacy of these different gene therapy strategies, ultimately paving the way for the development of targeted interventions for cardiac diseases.

## Figures and Tables

**Figure 1:**
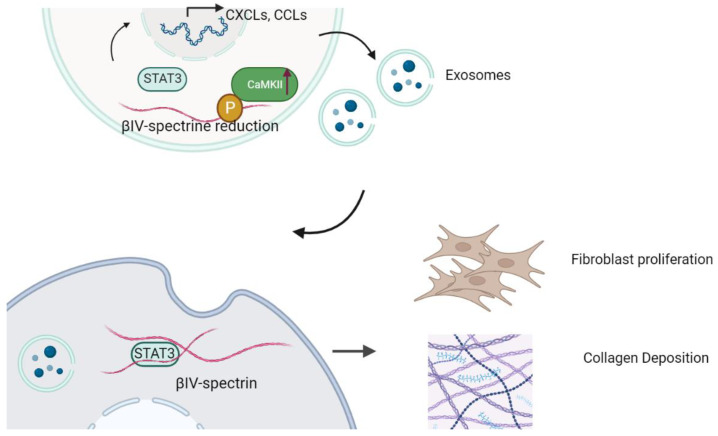
βIV-spectrin/CaMKII/STAT3 signalosome regulates fibroblast–fibroblast communication and fibrosis. The reduction in βIV-spectrin caused by ablation or CaMKII-mediated degradation promotes STAT3 translation into the nucleus. βIV-spectrin forms a complex with STAT3 and CaMKII, which directly phosphorylate βIV-spectrin. CaMKII: Calcium/calmodulin-dependent protein kinase II. STAT3: signal transducer and activator of transcription 3 (STAT3).

**Figure 2:**
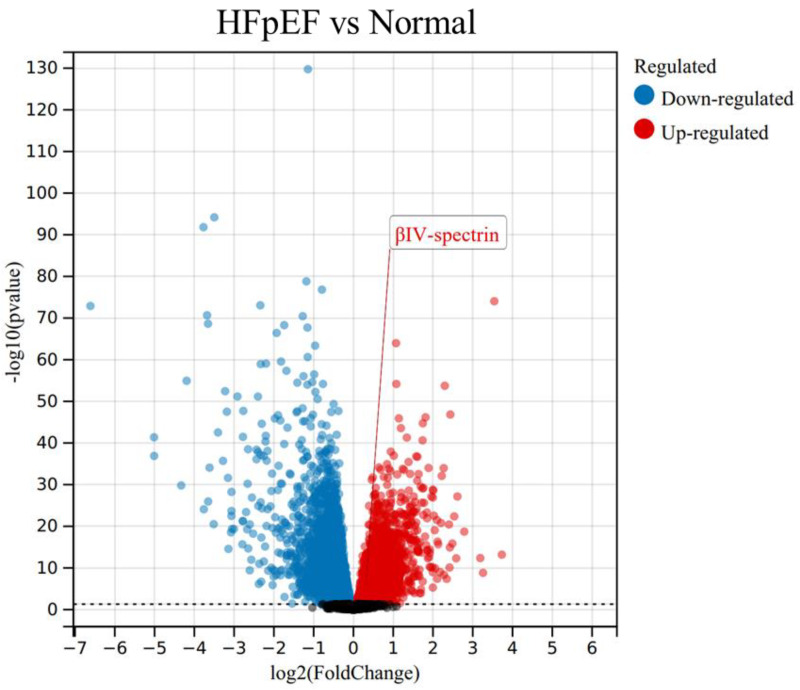
Dysregulation of βIV-spectrin in heart failure with preserved ejection fraction (HFpEF) (GSE3586).
